# Hearing loss and its link to cognitive impairment and dementia

**DOI:** 10.3389/frdem.2023.1199319

**Published:** 2023-06-15

**Authors:** Abdul Azeem, Arun Julleekeea, Beth Knight, Isha Sohail, Michael Bruyns-Haylett, Magdalena Sastre

**Affiliations:** ^1^Department of Brain Sciences, Imperial College London, London, United Kingdom; ^2^Department of Bioengineering, Imperial College London, London, United Kingdom

**Keywords:** hearing loss, dementia, Alzheimer's disease, hearing aid, cochlear implant, models of hearing loss, cognitive impairment

## Abstract

Hearing loss is an important risk factor for the development of dementia, particularly Alzheimer's disease (AD). Mid-life hearing loss increases the risk of developing dementia by double any other single factor. However, given this strong connection between hearing loss and AD, the mechanisms responsible for this link are still unknown. Data from observational studies relating hearing loss and cognitive impairment, measured with standardized questionnaires, has shown a strong relationship between them. Similar findings have emerged from animal studies, showing that the induction of hearing loss via prolonged loud sound exposure or ear canal blocking, can impair cognitive abilities. Interestingly, patients with age-related hearing impairment exhibit increased phosphorylated tau in the cerebrospinal fluid, but no such relationship has been identified for amyloid-β. In addition, hearing loss predisposes to social isolation precipitating the development of dementia through a supposed reduction in cognitive load and processing requirements. Given this link between hearing loss and dementia, the question arises whether the restoration of hearing might mitigate against the onset or progress of AD. Indeed, there is a growing body of research that suggests that those who wear hearing aids for age-related hearing problems maintain better cognitive function over time than those who do not. These are compelling findings, as they suggest the use of hearing aids has the potential to be a cost-effective treatment for those with hearing loss both prior (for those at high risk for AD) and after the development of symptoms. This review aims to summarize the current theories that relate hearing loss and cognitive decline, present the key findings of animal studies, observational studies and summarize the gaps and limitations that need to be addressed in this topic. Through this, we suggest directions for future studies to tackle the lack of adequately randomized control trials in the field. This omission is responsible for the inability to provide a conclusive verdict on whether to use hearing interventions to target hearing-loss related cognitive decline.

## 1. Introduction

Dementia poses a global burden—a 2022 report estimated that in 2019, 55 million people were living with a diagnosis of dementia (Gauthier et al., 2022). Future projections suggest that dementia will continue to increase in prevalence, approaching 140 million individuals by 2050 (Gauthier et al., 2022). To slow this exponential growth, novel interventions, either improving management of those already diagnosed, or via the prevention of those at an increased risk of developing it, are imperative.

Logically, risk factors for impaired cognition precede those for dementia, due to its clinical progression. Notably, age is distinguished as an important risk factor—the natural aging process leads to an inherent risk of cognitive decline, independent of dementia. Indeed, aging itself is simultaneously associated with a 70% increased incidence of dementia (Juan and Adlard, 2019). In addition, it is well established that loneliness and social isolation are contributing factors in poor cognitive performance, and are associated with an increased rate of decline (Cacioppo and Hawkley, 2009; Dominguez et al., 2021). Further to this, research has shown that individuals categorized as socially isolated have a 50% increased relative risk of developing dementia (Evans et al., 2018; Dominguez et al., 2021). Interestingly, a recent Lancet review (Livingston et al., 2020) focusing on various modifiable risk factors for the development of dementia, showed the importance of risk reduction, suggesting that modifying such risk factors has the potential to reduce the likelihood of dementia by 40% (LaPlume et al., 2022). Among different risks factors, including traumatic brain injury, hypertension, depression, and diabetes mellitus, the review highlights hearing loss (HL) as one of the potential factors that, when excluded, reduced the risk of dementia by 8%. Mild, moderate or severe HL particularly in the middle-life (specified as between the ages of 45 and 65) has been associated with an increase of 2, 3 and 5 times respectively in the risk of reduced cognition and dementia (Lin et al., 2011; Livingston et al., 2020). Furthermore, observational studies suggest that the severity of hearing impairment (HI) is associated with a risk of accelerated cognitive decline (LaPlume et al., 2022), and one report even demonstrated that the dementia risk increased linearly with the severity of baseline HL (1.27 per 10 dB loss) (Ford et al., 2018). However, limitations remain regarding confounding factors, which may influence the generalizability of these findings (Ford et al., 2018).

In this review, we explore HL as a risk factor for the development of cognitive impairment and dementia, focusing on molecular mechanisms. Through this, we summarize the research surrounding clinical manifestations of HL in dementia, proposed mechanisms to this relationship, and the role of interventions, including the use of hearing aid (HA) devices to minimize the effects of HL in the progression of cognitive impairment to dementia. We highlight the importance of further understanding the relationships between the proposed mechanisms and cognitive impairment, with the eventual aim of providing effective novel interventions to mitigate the risk of HL in the development of dementia.

## 2. Causes of hearing loss and their link to dementia

Hearing loss affects currently 466 million people Worldwide (World Health Organisation, 2021). Individuals suffering from disabling HL account for over 5% of the global population (World Health Organisation, 2021) and it is predicted that by 2050, nearly 1 in every 10 people will require hearing rehabilitation. Causative factors of hearing loss include genetic factors, ear infections, cerumen impaction (impacted ear wax), trauma to the ear or head, loud noise/loud sounds (NHL), ototoxic medicines and others.

HL mechanisms implicated in dementia predominantly surround sensorineural hearing changes, in which there is dysfunction of the cochlea. Most often, it is seen as age-related hearing loss (ARHL) or presbycusis, which affects around 40% of individuals over the age of 65 (Gates and Mills, 2005). Causes of HL including presbycusis, noise-induced hearing loss (NIHL) and ototoxicity, all precipitate permanent HL, and subsequently, result in limited management options (Lee and Bance, 2019).

ARHL is highly polygenic, with over 100 genes known to underlie human non-syndromic hearing impairment (Lewis et al., 2018; Van Camp and Smith, 2023), and of these many genes, possibly each makes small contributions to create an estimated heritability of 36–70% (Nagtegaal, 2019). Most cases of genetic deafness imply alterations of the cochlea, the auditory sensory organ; for instance, the OTOF gene encodes the protein otoferlin, which allows synaptic vesicles to fuse to the plasma membrane in the ribbon synapse. Thus, mutations in this gene can lead to a deficiency in exocytosis in the inner hair cells, which interrupts auditory signal transmission and can cause prelingual deafness (Vona et al., 2020). However, some forms of genetic hearing loss can involve failures in the central auditory system. Genome wide association studies (GWAS) that have focused on the genes associated with ARHL, although showing some overlap, do not show strong agreement with one another (Nagtegaal, 2019; Wells et al., 2019; Liu et al., 2021; Lewis et al., 2022). When considering a genetic relationship between hearing loss and AD, no GWAS have found a direct causal link between ARHL and AD. However, a recent study using UK biobank data (Brenowitz et al., 2020) demonstrated that a genetic risk for AD also influences speech-in-noise hearing. In addition, in another report (Mitchell et al., 2020), individuals with higher polygenic risk score (PRS) for AD were more likely to experience hearing difficulty than those with lower PRS.

Sudden sensorineural hearing loss (SSHL) has also been identified as a risk factor for the development of dementia. This form of HL is characterized as an otological emergency, defined by HL of at least 30 dB over 72 h, affecting at least three different auditory frequencies, often with a viral, vascular, or autoimmune etiology (Lee and Bance, 2019). In a retrospective cohort study comparing the incidence of dementia in individuals with and without a previous diagnosis of SSHL, it was established that the occurrence of SSHL was associated with a 1.39 times higher likelihood in the incidence of all-cause dementia (Tai et al., 2021).

**Figure 1:**
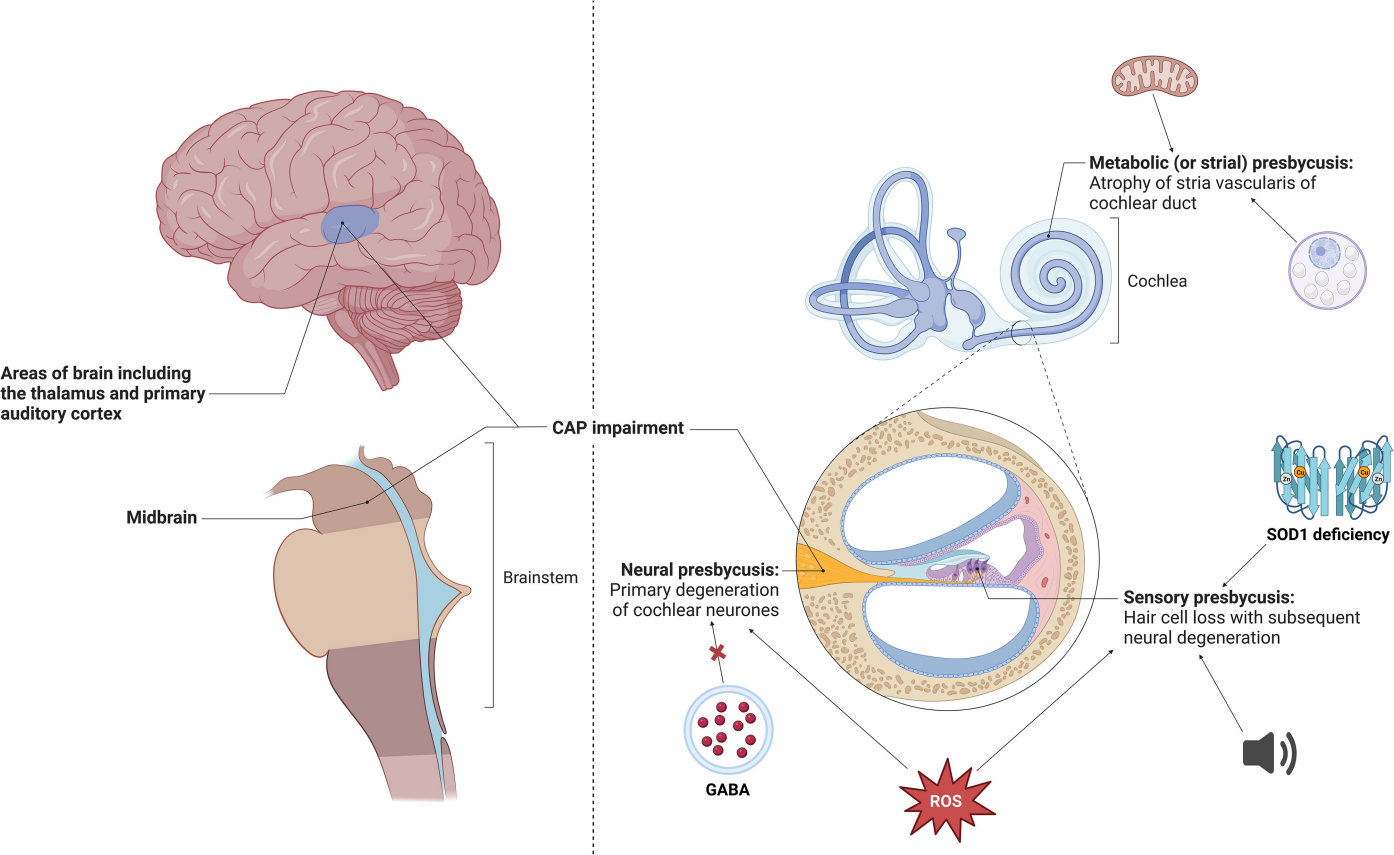
Different types of age-related hearing impairment, including atrophy of the stria vascularis, hair cell loss and primary cochlear neuron degeneration (Quaranta et al., 2015; Fortunato et al., 2016). Specific changes seen in stria vascularis include age-related morphological changes such as numerous cytoplasmic vacuoles, enlargement of intracellular spaces and irregularity of mitochondria and its constituents, most notably disorganization of cristae (Lyu et al., 2020). These changes have been shown to be precipitated by oxidative damage and downregulation of TMEM16A, a calcium-activated chloride channel (Spicer and Schulte, 2005; Zhou et al., 2019). Cochlear disease or trauma have been shown to give rise to hair cell loss, with potential causative factors including continuous industrial noise exposure, reactive oxygen species and Superoxide dismutase (SOD1) deficiency (McFadden et al., 1999a,b, 2001; Emmerich et al., 2000; Huang et al., 2000; Vlajkovic and Thorne, 2021). Primary neural degeneration via disconnecting of auditory neurones from their hair cell targets during aging has been shown to trigger the loss of hair cells (Viana et al., 2015; Wu et al., 2019). Studies have identified that loss of GABA in the central nucleus of the inferior colliculus and the build-up of reactive oxygen species has led to neural presbycusis (Caspary et al., 1990, 2013; Huang et al., 2000). Made using BioRender software. CAP, central auditory processing; ROS, reactive oxygen species.

However, specifically within presbycusis, a variety of other mechanisms have also been implicated, including metabolic factors (for instance, mitochondrial dysfunction), oxidative stress (including changes in reactive oxygen species and Superoxide dismutase deficiency), neurotransmitter imbalance (GABA deficiency), among others (Jafari et al., 2021) (Figure 1). Further insight into the underlying mechanisms and how these different classes of ARHL may herald dementia, is not yet completely understood (Bowl and Dawson, 2019).

## 3. Current understanding of the association between dementia and hearing loss

Poorer scores on the Mini Mental State Examination (MMSE) have been shown to strongly correlate with deficits in audiological testing (Quaranta et al., 2015; Golub et al., 2019; Saji, 2021; Mohammed et al., 2022; Huang et al., 2023). This link is supported by the demonstration of a relationship between HL and MRI brain atrophy (Jafari et al., 2021). Despite a recent study suggesting otherwise (Marinelli et al., 2022), meta-analyses pooling observational studies have further strengthened evidence of an association between ARHL and cognitive decline (Loughrey et al., 2017; Mamo et al., 2018; Liang et al., 2021). Similar correlations between age-associated hearing loss and cognitive decline have been reported in mice (Dong et al., 2018). However, this observational data should be interpreted with caution, as there is a subsequent inability to draw causal links. Additionally, potential confounders that are often seen in the elderly, such as nutritional issues, visual impairment, vascular risk factors, frailty, bad physical health, depression, and other mental illnesses, could contribute to the trend seen (Hirose et al., 2014; Gill et al., 2020). Among those, the weakening of the vascular system is an interesting link, since it is involved in both deafness and neurodegeneration. Vascular networks of the cochlea (Kurata et al., 2016; Nyberg et al., 2019) and the auditory cortex might be impaired in genetic forms of hearing loss, although neural activity by itself can also affect the remodeling of the vascular system (Lacoste et al., 2014; Whiteus et al., 2014).

Interestingly, central auditory pathway disorder in the studied cohorts correlated more with cognitive changes than non-central HL. Why this occurs is not completely understood, but it is hypothesized that the dementia-related neurodegeneration and healthy aging could affect auditory areas of the brain, and lead to subsequent HI (Bidelman et al., 2014; Johnson et al., 2021). Age-related hearing problems are common among people with dementia and are associated with poor cognitive function and reduced quality of life (Maharani et al., 2018a), suggesting that sensory markers could be useful to detect and target cognitive aging.

Contrastingly, there could be an indirect mechanism, as HI predisposes individuals to social isolation and subsequent dementia risk (Sardone et al., 2020). Both dementia and HL present highly heterogeneous conditions, and therefore the investigation of various subtypes within each could be valuable to help further the field of research exploring their interrelationship. Different forms of both dementia and hearing loss may have different trajectories and studies to elucidate this would allow more effective treatments, as shown in frailty (Segaux et al., 2019).

Mirroring human studies, investigations with animal models of AD, including 5XFAD, APP/PS1 and 3XTg transgenic mice have demonstrated both significant HL and degeneration of spiral ganglion cells in the cochlea (Wang and Wu, 2015, 2021; O'Leary et al., 2017; Liu et al., 2020; Weible et al., 2020). Additionally, 5XFAD mice subjected to surgical deafness through tympanic membrane resection i.e., conductive hearing loss (CHL), showed greater cognitive impairment than those with no HI (Kim et al., 2020). This could imply a reciprocal relationship—that AD can lead to auditory deficits and auditory deficits can influence cognition. Whether these AD models can be applied to dementia generally, is not yet known.

## 4. Mechanisms underlying hearing loss, cognitive function, and AD pathology in animal models

Animal models attempting to elucidate the relationship between dementia and HL center around rodents with induced hearing deficits. Two main paradigms are currently utilized to induce these deficits. Most common methods applied expose rodents to high-volume background noise for several days, inducing permanent trauma (Wang and Wu, 2015; Zhuang et al., 2020; Kurioka et al., 2021a; Li et al., 2021; Paciello et al., 2021), or by the bilateral cochlear ablation. Alternatively, HI is mechanically modeled through insertion of a silicone mold into the ear canal, followed by permanent suturing to close the canal (Paciello et al., 2021). Additionally, administration of ototoxic substances such as furosemide and kanamycin can be employed to induce sensorineural HL, but this is not used often (Shen et al., 2021). These methods inflict physical trauma and stress and subsequently, could influence cognition via mechanisms independent of HI, confounding results. Following HL induction, mice undergo acoustic brainstem response testing, a quantitative method of measuring inner ear and hearing pathway responses to sound (Zhuang et al., 2020; Li et al., 2021). Animal studies more specifically looking at brain region histology conduct further posthumous histology of the animal brain (Li et al., 2021).

One of the key mechanisms explored through animal models and human cases is how, following HL, subsequent decline in hippocampal neurogenesis can lead to cognitive deterioration (Kurioka et al., 2021b). The hippocampus, primarily involved in memory and auditory information processing, is one of the key areas affected in dementia. Interestingly, studies in a rat model with hearing loss and/or Aβ administration demonstrated that the group with lower cognitive abilities was the one with both amyloid-beta (Aβ) and SSHL (Chang et al., 2019). In this report, it was demonstrated a significant decrease in hippocampal synaptic proteins in the Aβ-deaf group, implying that HL influences synaptic plasticity, and that there must be a connection between the central auditory cortex and the hippocampus (Chang et al., 2019).

Another potential link between hearing loss and cognitive impairment in animals implies changes in neuroinflammatory markers. Two animal model studies examining NIHL demonstrated alterations in microglia and the presence of irregularly shaped somas when compared to the control mice (Zhuang et al., 2020; Li et al., 2021). Similar results were reported in a conductive hearing loss (CHL) animal model, showing morphological microglial changes specifically in the dentate gyrus and subgranular zone (Kurioka et al., 2021b). Therefore, it appears that microglial activation does occur following HL and could cause a consequent impairment of hippocampal neurogenesis. However, recent studies seem to contradict these findings, showing that there is no correlation between spatial learning ability and the level of hearing loss or altered microglial density in the hippocampus following noise exposure in rats, suggesting that other mechanisms are involved in the hippocampal-dependent cognitive dysfunction due to noise exposure (Patel et al., 2022). Besides changes in glial cell activation, other studies implementing hearing loss by the use of toxins in mouse models of neurodegeneration have shown increases in inflammatory cytokines such as IL-1β and TNF-α (Ren et al., 2011; Shen et al., 2021).

There are also evidences from human and preclinical studies that AD pathological hallmarks, including hyperphosphorylated Tau (p-Tau) and Aβ, could be affected by HL. A recent report in C57Bl6 in mice exposed to noise induced hearing loss have shown reduced cognition and p-tau and lipofuscin in the hippocampus (Park et al., 2018). Another study in rats also demonstrated that chronic noise exposure (CNE) was associated with tau hyperphosphorylation in the hippocampus and the prefrontal cortex (Cui et al., 2012). In agreement with this, human studies have associated ARHL with higher CSF levels (and accelerated rates of elevation) of p-Tau, but have demonstrated no association between ARHL and the levels of Aβ_42_ in CSF (Xu et al., 2019; Sarant et al., 2022). In line with this, a cross-sectional cohort study utilizing positron emission tomography scans to investigate the presence of Aβ, also found no association between HL and Aβ load (Sarant et al., 2022). In contrast, studies in rats have shown that chronic exposure to noise resulted in increased generation of endogenous Aβ levels in hippocampus (Cui et al., 2015).

In line with reports showing alterations in hippocampal function, HL has been involved in changes on neurotransmitter expression as well. ARHL mouse models have shown changes in NMDA receptor expression within the hippocampus, which can take place after 4 months of natural HL (Cui et al., 2009). Additionally, GABAa and GABAb receptors were altered during this time period. The change in these neurotransmitter receptors is implicated in hippocampal synaptic plasticity, affecting hippocampal learning, and resulting in cognitive decline (Guo et al., 2021).

An emerging hypothesis links social memory impairment and HL—partially due to limitations of social interaction faced by those with a HI (Beckmann et al., 2020). A study using a mouse model of congenital deafness with OTOF gene knockout, attempted to investigate this through eliminating hearing input from the cochlea to the brain, resulting in decreased social memory. If this hypothesis is correct, it would help explain why associated brain regions' functionality, for example, the hippocampus, is affected following HL (Glick and Sharma, 2020). Furthermore, there is the possibility of a compensatory mechanism, whereby the brain may offset certain neural networks to promote better hearing. In line with this, a human MRI study of HI individuals demonstrated that auditory brain regions displayed an increase in activity, whilst other areas showed a consequential decrease (Rigters et al., 2017). Therefore, this could indicate that this activity decrease is linked to the reduction in cognitive function seen in HL.

## 5. Clinical interventions and their limitations

As discussed, observational studies have established an association between HI and an increased risk of accelerated cognitive decline. The emphasis placed on HL as a modifiable risk factor for dementia (LaPlume et al., 2022) has led to the reasonable hypothesis that audibility restoration could potentially alleviate cognitive decline, by counteracting the postulated mechanisms which underlie this process and by reducing social isolation, loneliness, and depressed mood (Jiang et al., 2023). It is hypothesized that hearing restoration, may reverse cognitive changes of cortical reallocation of sensory processing patterns, leading to consequent cognitive gains (Glick and Sharma, 2020).

In two short-term rodent studies modeling CHL through use of ear plugs, significant cochlear degeneration (Kurioka et al., 2021b) and changes in the neurotransmitter expression within auditory neurons (Kurioka et al., 2020) was demonstrated following reduced auditory inputs. Interestingly, removal of the ear plugs led to recovery of almost all these anatomical changes, suggesting that similar treatment with either HAs or cochlear implantation in humans could reverse such changes, and perhaps cognitive processing.

Overall, many observational, prospective and retrospective cohort, cross-sectional and longitudinal studies have linked HA use to lower rates of cognitive decline (Maharani et al., 2018b; Glick and Sharma, 2020; Cuoco et al., 2021; Fernandes and Mastroianni Kirsztajn, 2021; Sugiura et al., 2021, 2022; Bucholc et al., 2022; Dillard et al., 2022; Vella Azzopardi et al., 2023) and dementia (Maharani et al., 2018b; Mahmoudi et al., 2019; Byun et al., 2022; Dillard et al., 2022; Naylor et al., 2022). Recent data from the UK biobank support these findings, showing no increased risk of dementia in people with hearing loss using hearing aids (Jiang et al., 2023). Strikingly, one of the studies demonstrated that the diagnosis of dementia is associated with a 54% subsequent reduction in HA usage compared to those without dementia (Naylor et al., 2022). The importance is thus placed on not only increasing HA usage amongst older individuals at higher risk of both HL and dementia, but also working to maximize adherence in this community.

On the other hand, two prospective longitudinal studies found no statistical significance between cognitive scores pre- and post- HA fittings (Sarant et al., 2020; Kawade et al., 2022). Furthermore, the only double-blind, randomized controlled trial evaluating the cognitive benefit of HAs in patients with AD also found no statistically significant relationship (Nguyen et al., 2017). There is a clear lack of consensus of the current evidence, but this could be explained by the various limitations and differences across all the study designs.

Generally, studies investigating cognition pre- vs. post- cochlear implantation reported either no change (Sonnet et al., 2017; Kramer et al., 2018; Sarant et al., 2019), an improvement (Jayakody et al., 2017; Claes et al., 2018; Mosnier et al., 2018; Völter et al., 2021, 2022a,b; Calvino et al., 2022), or a mixed picture across the various subtests (Sorrentino et al., 2020; Huber et al., 2021). Cochlear implantation is one phase of a complex rehabilitation process involving multiple appointments and training programmes following surgery (British Cochlear Implant Group, 2023). These all pose opportunities for increased engagement in cognitive stimulation, known to reduce risk of cognitive decline. Whether the cognitive improvement seen across these studies is due to restored hearing itself or a confounding influence of rehabilitation, would be difficult to determine (Völter et al., 2022b).

Much of the heterogeneity across both the hearing aids and cochlear implantation literature exists due to the variety of tests used to measure cognitive function. Some papers choose to use standardized screening tests like the MMSE (Sonnet et al., 2017; Sorrentino et al., 2020; Herzog et al., 2022; Vella Azzopardi et al., 2023), or the MoCA (Cuoco et al., 2021; Fernandes and Mastroianni Kirsztajn, 2021) as an assessment of global cognitive function, whilst other studies focus on assessing subdomains of cognition through use of investigations like the ALAcog (Sarant et al., 2019; Völter et al., 2021; Calvino et al., 2022), ADAScog (Nguyen et al., 2017), Cogstate (Sarant et al., 2019, 2020), RBANS-H (Claes et al., 2018; Brewster et al., 2020; Calvino et al., 2022) and various other subtests (Jayakody et al., 2017; Mosnier et al., 2018; Füllgrabe, 2020; Kurioka et al., 2020). Using tests like the MMSE, which utilize auditory assessments, leaves HI individuals at a direct disadvantage. Cognition prior to rehabilitation could be underestimated, and treatment may simply allow the participant to perform better due to increased ability to understand the task presented (Füllgrabe, 2020). A ceiling effect is also observed through use of MMSE (Sonnet et al., 2017)—those with a normal cognition pre-intervention are unable to show any further improvement. The introduction of a standardized neurocognitive battery, which assesses all necessary subdomains and is adapted for the HI, needs to be implemented to allow comparison across the literature.

Further sources of heterogeneity across results could be due to the varying durations of studies. Undoubtedly, measures of cognitive function are susceptible to time due to the cognitive decline observed with aging. Some studies examined cognitive outcomes over longer periods, ranging from 3.5 to 18 years (Maharani et al., 2018b; Naylor et al., 2022; Sugiura et al., 2022), whilst others observed significantly shorter periods; for example, one report (Glick and Sharma, 2020) studied participants over 6 months, whilst another (Fernandes and Mastroianni Kirsztajn, 2021) over only 12 weeks. Shorter trials should be interpreted with additional caution—interventions may not have significant time to influence a change, nor do they consider the normal cognitive decline represented over time.

## 6. Conclusions

Overall, a clear trend between HL and dementia is demonstrated across the literature. However, evidence is largely composed of observational studies and there remains a lack of interventional studies relating HL to dementia. This is due to a clinical dilemma- that it would be unethical to deny treatment to those with HL, especially when considering the ramifications of untreated HL, not just in relation to dementia. The introduction of open label trials, recruiting from surgical candidates for cochlear implantation, could circumvent this. These trials could build on previous limitations by including a standardized neurocognitive battery. Furthermore, despite a multitude of animal models demonstrating potential underlying mechanisms linking HI and cognitive decline, further research, particularly with longer follow-up periods and invasive recording technology is required to help interrogate the mechanisms of action.

Uncertainties also remain in the implementation of HAs and CIs as interventions against cognitive decline. As a result, algorithms and guidelines may need to be synthesized from future interventional studies to best improve patient outcomes. There should be a focus on maximizing early HL diagnoses and swift implementation of auditory treatment to mitigate the risks of cognitive decline. Models calculating dementia risk would allow the stratification of the geriatric population and perhaps help clinicians decide risk and subsequent necessary auditory treatment. Additionally, technology such as smart phone connected HAs could target the current limitations encompassing HA usage in those with dementia.

## Author contributions

AA wrote the current understanding of the association between dementia and hearing loss. AJ wrote the introduction and the most of the section of causes of hearing loss and dementia and made the figure. BK wrote the clinical interventions and their limitations. IS wrote the mechanisms underlying hearing loss, cognitive function, and AD Pathology in animal models. MB-H and MS designed, edited the manuscript and organized the references. All authors contributed to the article and approved the submitted version.

## References

[B1] Beckmann D. Feldmann M. Shchyglo O. Manahan-Vaughan D. (2020). Hippocampal synaptic plasticity, spatial memory, and neurotransmitter receptor expression are profoundly altered by gradual loss of hearing ability. Cereb. Cort. 30, 4581–4596. 10.1093/cercor/bhaa061 32202614 PMC7325716

[B2] Bidelman G. M. Villafuerte J. W. Moreno S. Alain C. (2014). Age-related changes in the subcortical-cortical encoding and categorical perception of speech. Neurobiol. Aging 35, 2526–40. 10.1016/j.neurobiolaging.2014.05.006 24908166

[B3] Bowl M. R. Dawson S. J. (2019). Age-Related Hearing Loss. Cold Spring Harbor Perspect. Biol. 9, a033217. 10.1101/cshperspect.a033217 30291149 PMC6671929

[B4] Brenowitz W. D. Filshtein T. J. Yaffe K. Walter S. Ackley S. F. Hoffmann T. J.. (2020). Association of genetic risk for Alzheimer disease and hearing impairment. Neurology 95, e2225. 10.1212/WNL.0000000000010709 32878991 PMC7713783

[B5] Brewster K. K. Pavlicova M. Stein A. Chen M. Chen C. Brown P. J.. (2020). A pilot randomized controlled trial of hearing aids to improve mood and cognition in older adults. Int. J. Geriatric Psychiatry. 35, 842–850. 10.1002/gps.5311 32291802 PMC7656495

[B6] British Cochlear Implant Group (2023). Rehabilitation. Available online at: https://www.bcig.org.uk/assessed/rehabilitation/ (accessed 2023).

[B7] Bucholc M. Bauermeister S. Kaur D. McClean P. L. Todd S. (2022). The impact of hearing impairment and hearing aid use on progression to mild cognitive impairment in cognitively healthy adults: an observational cohort study. Alzheimer's Dement. 8, e12248. 10.1002/trc2.12248 35229022 PMC8863441

[B8] Byun H. Chung J. H. Lee S. H. Kim E. M. Kim I. (2022). Dementia in a hearing-impaired population according to hearing aid use: a nationwide population-based study in Korea. Ear Hear. 43, 1661–1668. 10.1097/AUD.0000000000001249 35671072 PMC9592173

[B9] Cacioppo J. T. Hawkley L. C. (2009). Perceived social isolation and cognition. Trends Cogn. Sci. 13, 447–454. 10.1016/j.tics.2009.06.005 19726219 PMC2752489

[B10] Calvino M. Sánchez-Cuadrado I. Gavilán J. Gutiérrez-Revilla M. A. Polo. R. Lassaletta L. (2022). Effect of cochlear implantation on cognitive decline and quality of life in younger and older adults with severe-to-profound hearing loss. Eur. Arch. Otorhinolaryngol. 279,4745–4759. 10.1007/s00405-022-07253-6 35044508 PMC9474541

[B11] Caspary D. M. Hughes L. F. Hughes L. L. (2013). Age-related GABAA receptor changes in rat auditory cortex. Neurobiol. Aging 34, 1486–1496. 10.1016/j.neurobiolaging.2012.11.009 23257264 PMC3570724

[B12] Caspary D. M. Raza A. Lawhorn Armour, B. A. Pippin J. Arneric S. P. (1990). Immunocytochemical and neurochemical evidence for age-related loss of GABA in the inferior colliculus: implications for neural presbycusis. J. Neurosci. 10, 2363–2372. 10.1523/JNEUROSCI.10-07-02363.1990 1973948 PMC6570369

[B13] Chang M. Kim H. J. Mook-Jung I. Oh S. (2019). Hearing loss as a risk factor for cognitive impairment and loss of synapses in the hippocampus. Behav. Brain Res. 372, 112069. 10.1016/j.bbr.2019.112069 31271817

[B14] Claes A. Van de Heyning P. Gilles A. Van Rompaey V. Mertens G. (2018). Cognitive performance of severely hearing-impaired older adults before and after cochlear implantation: preliminary results of a prospective, longitudinal cohort study using the RBANS-H. Otol. Neurotol. 39, e765–e773. 10.1097/MAO.0000000000001936 30153132

[B15] Cui B. Li K. Gai Z. She X. Zhang N. Xu C.. (2015). Chronic noise exposure acts cumulatively to exacerbate Alzheimer's disease-like Amyloid-β pathology and neuroinflammation in the rat hippocampus. Sci Rep. 5,12943. 10.1038/srep12943 26251361 PMC4528219

[B16] Cui B. Wu M. She X. (2009). Effects of chronic noise exposure on spatial learning and memory of rats in relation to neurotransmitters and NMDAR2B alteration in the hippocampus. J. Occup. Health 51,152–8. 10.1539/joh.L8084 19225220

[B17] Cui B. Zhu L. She X. Wu M. Ma Q. Wang T.. (2012). Chronic noise exposure causes persistence of tau hyperphosphorylation and formation of NFT tau in the rat hippocampus and prefrontal cortex. Exp Neurol. 238, 122–9. 10.1016/j.expneurol.2012.08.028 22971273

[B18] Cuoco S. Cappiello A. Scarpa A. Troisi D. Autuori M. Ponticorvo S.. (2021). Neuropsychological profile of hearing-impaired patients and the effect of hearing aid on cognitive functions: an exploratory study. Sci. Rep. 11, 9384. 10.1038/s41598-021-88487-y 33931670 PMC8087665

[B19] Dillard L. K. Pinto A. Mueller K. D. Schubert C. R. Paulsen A. J. Merten N.. (2022). Associations of hearing loss and hearing aid use with cognition, health-related quality of life, and depressive symptoms. J. Aging Health. 10.1177/08982643221138162 36412130 PMC10200823

[B20] Dominguez L. J. Veronese N. Vernuccio L Catanese G. Inzerillo F. Salemi G.. (2021). Nutrition, physical activity, and other lifestyle factors in the prevention of cognitive decline and dementia. Nutrients 13, 4080. 10.3390/nu13114080 34836334 PMC8624903

[B21] Dong Y. Guo C. Chen D. Chen S. Peng Y. Song H.. (2018). Association between age-related hearing loss and cognitive decline in C57BL/6J mice. Mol. Med. Rep. 18, 1726–1732. 10.3892/mmr.2018.9118 29901198

[B22] Emmerich E. C. Richter F. G. Reinhold U. Linss V. Linss W. (2000). Effects of industrial noise exposure on distortion product otoacoustic emissions (DPOAEs) and hair cell loss of the cochlea – long term experiments in awake guinea pigs. Hear. Res. 148, 9–17. 10.1016/S0378-5955(00)00101-5 10978821

[B23] Evans I. E. M. Llewellyn D. J. Matthews F. E. Woods R. T. Brayne C. Clare L. (2018). Social isolation, cognitive reserve, and cognition in healthy older people. PLoS ONE 13, e0201008. 10.1371/journal.pone.0201008 30118489 PMC6097646

[B24] Fernandes D. E. Mastroianni Kirsztajn G. Almeida, K. (2021). Effect of hearing aids on attention, memory, and auditory evoked potentials: a pragmatic, single-blinded, and randomised pilot clinical trial. Int. J. Clin. Pract. 75, e13953. 10.1111/ijcp.13953 33345388

[B25] Ford A. H. Hankey G. J. Yeap B. B. Golledge J. Flicker L. Almeida O.P. (2018). Hearing loss and the risk of dementia in later life. Maturitas 112, 1–11. 10.1016/j.maturitas.2018.03.004 29704910

[B26] Fortunato S. Forli F. Guglielmi V. De Corso E. Paludetti G. Berrettini S.. (2016). A review of new insights on the association between hearing loss and cognitive decline in ageing. Acta Otorhinolaryngol. Italica. 36, 155–166. 10.14639/0392-100X-993 27214827 PMC4977003

[B27] Füllgrabe C. (2020). On the possible overestimation of cognitive decline: the impact of age-related hearing loss on cognitive-test performance. Front. Neurosci. 14, 454. 10.3389/fnins.2020.00454 32581666 PMC7296091

[B28] Gates G. A. Mills J.H. (2005). Presbycusis. Lancet 366, 1111–1120. 10.1016/S0140-6736(05)67423-5 16182900

[B29] Gauthier S. Webster C. Servaes S. Morais J. A. Rosa-Neto P. (2022). World Alzheimer Report 2022: Life After Diagnosis: Navigating Treatment, Care and Support. London: Alzheimer's Disease International.

[B30] Gill T. M. Han L. Gahbauer E. A. Leo-Summers L. Murphy T. E. (2020). Risk factors and precipitants of severe disability among community-living older persons. JAMA Network Open 3, e206021. 10.1001/jamanetworkopen.2020.6021 32484551 PMC7267844

[B31] Glick H. A. Sharma A. (2020). Cortical neuroplasticity and cognitive function in early-stage, mild-moderate hearing loss: evidence of neurocognitive benefit from hearing aid use. Front. Neurosci.14, 93. 10.3389/fnins.2020.00093 32132893 PMC7040174

[B32] Golub J. S. Brickman A. M. Ciarleglio A. J. Schupf N. Luchsinger J.A. (2019). Association of subclinical hearing loss with cognitive performance. Arch. Otolaryngol. Head Neck Surg. 146, 57–67. 10.1001/jamaoto.2019.3375 31725853 PMC6865840

[B33] Guo R. Li Y. Liu J. Gong S. Liu K. (2021). Complete elimination of peripheral auditory input before onset of hearing causes long-lasting impaired social memory in mice. Front. Neurosci. 15, 723658. 10.3389/fnins.2021.723658 34385906 PMC8353330

[B34] Herzog J. A. Buchman C. A. Kallogjeri D. Chen S. Wick C. Durakovic N.. (2022). Cognitive assessment in elderly cochlear implant recipients: long-term analysis. Laryngoscope. 10.1002/lary.30466 36300628

[B35] Hirose T. Hasegawa J. Izawa S. Enoki H. Suzuki Y. Kuzuya M. (2014). Accumulation of geriatric conditions is associated with poor nutritional status in dependent older people living in the community and in nursing homes. Geriat. Gerontol. Int. 14, 198–205. 10.1111/ggi.12079 24118829

[B36] Huang A. R. Jiang K. Lin F. R. Deal J. A. Reed N. S. (2023). Hearing loss and dementia prevalence in older adults in the US. JAMA 329, 171–173. 10.1001/jama.2022.20954 36625819 PMC9856835

[B37] Huang T. Cheng A. G. Stupak H. Liu W. Kim A. Staecker H.. (2000). Oxidative stress-induced apoptosis of cochlear sensory cells: otoprotective strategies. Int. J. Dev. Neurosci. 18, 259–270. 10.1016/S0736-5748(99)00094-5 10715580

[B38] Huber M. Roesch S. Pletzer B. Lukaschyk J. Lesinski-Schiedat A. Illg A. (2021). Can cochlear implantation in older adults reverse cognitive decline due to hearing loss? Ear Hear. 42, 1560–1576. 10.1097/AUD.0000000000001049 34028233

[B39] Jafari Z. Kolb B. E. Mohajerani M. H. (2021). Age-related hearing loss and cognitive decline: MRI and cellular evidence. Ann. N.Y. Acad. Sci 1500, 17–33. 10.1111/nyas.14617 34114212

[B40] Jayakody D. M. Friedland P. Nel E. Martins R. Atlas M. Sohrabi H. (2017). Impact of cochlear implantation on cognitive functions of older adults: pilot test results. Otol. Neurotol. 38, e289–e295. 10.1097/MAO.0000000000001502 28806341

[B41] Jiang F. Mishra S. R. Shrestha N. Ozaki A. Virani S. S. Bright T. (2023) Association between hearing aid use all-cause cause-specific dementia: an analysis of the UK Biobank cohort. Lancet Public Health 8, e329. 10.1016/S2468-2667(23)00048-8 .

[B42] Johnson J. C. S. Marshall C. R. Weil R. S. Bamiou D. Hardy C. J. D. Warren J.D. (2021). Hearing and dementia: from ears to brain. Brain 144, 391–401. 10.1093/brain/awaa429 33351095 PMC7940169

[B43] Juan S. M. A. Adlard P. A. (2019). Ageing and cognition biochemistry and cell biology of ageing: part II clinical science. J. Robin Harris, Viktor I. Korolchuk. Springer 91, 107–122. 10.1007/978-981-13-3681-2_5 30888651

[B44] Kawade Y. Uchida Y. Sugiura S. Suzuki H. Shimono M. Ito E.. (2022). Relationship between cognitive domains and hearing ability in memory clinic patients: how did the relationship change after 6 months of introducing a hearing aid? Auris Nasus Larynx. 50, 343–350. 10.1016/j.anl.2022.09.005 36175261

[B45] Kim J. S. Lee H. Lee S. Lee H. S. Jeong Y. J. Son Y.. (2020). Conductive hearing loss aggravates memory decline in Alzheimer model mice. Front Neurosci. 14, 843. 10.3389/fnins.2020.00843 32903751 PMC7438902

[B46] Kramer S. Vasil K. J. Adunka O. F. Pisoni D. B. Moberly A. C. (2018). Cognitive functions in adult cochlear implant users, cochlear implant candidates, and normal-hearing listeners. Laryngoscope Invest. Otolaryngol. 3, 304–310. 10.1002/lio2.172 30186962 PMC6119791

[B47] Kurata N. Schachern P. A. Paparella M. M. Cureoglu S. (2016). Histopathologic evaluation of vascular findings in the cochlea in patients with presbycusis. JAMA Otolaryngol. Head Neck Surg. 142, 173–178. 10.1001/jamaoto.2015.3163 26747711

[B48] Kurioka T. Mogi S. Tanaka M. Yamashita T. (2021a). Activity-dependent neurodegeneration and neuroplasticity of auditory neurons following conductive hearing loss in adult mice. Cell. Mol. Neurobiol. 41, 31–42. 10.1007/s10571-020-00829-y 32180095 PMC11448668

[B49] Kurioka T. Mogi S. Yamashita T. (2021b). Decreasing auditory input induces neurogenesis impairment in the hippocampus. Sci. Rep. 11, 423. 10.1038/s41598-020-80218-z 33432038 PMC7801596

[B50] Kurioka T. Mogi S. Yamashita T. (2020). Transient conductive hearing loss regulates cross-modal VGLUT expression in the cochlear nucleus of C57BL/6 mice. Brain Sci. 10, 260. 10.3390/brainsci10050260 32365514 PMC7287693

[B51] Lacoste B. Comin C. H. Ben-Zvi A. Kaeser P. S. Xu X. Costa Lda F.. (2014). Sensory-related neural activity regulates the structure of vascular networks in the cerebral cortex. Neuron 83, 1117–30. 10.1016/j.neuron.2014.07.034 25155955 PMC4166422

[B52] LaPlume A. A. McKetton L. Levine B. Troyer A.K. (2022). The adverse effect of modifiable dementia risk factors on cognition amplifies across the adult lifespan. Alzheimer's Dementia. 14, e12337. 10.1002/dad2.12337 35845262 PMC9277708

[B53] Lee J. W. Bance M.L. (2019). Hearing loss. Pract. Neurol. 19, 28–35. 10.1136/practneurol-2018-001926 30185631

[B54] Lewis M. A. Nolan L. S. Cadge B. A. Matthews L. J. Schulte B. A. Dubno J. R.. (2018). Whole exome sequencing in adult-onset hearing loss reveals a high load of predicted pathogenic variants in known deafness-associated genes and identifies new candidate genes. BMC Med. Genom. 11, 77. 10.1186/s12920-018-0395-1 30180840 PMC6123954

[B55] Lewis M. A. Schulte B. A. Dubno J. R. Steel K. P. (2022). Investigating the characteristics of genes and variants associated with self-reported hearing difficulty in older adults in the UK Biobank. BMC Biol. 20, 150. 10.1186/s12915-022-01349-5 35761239 PMC9238072

[B56] Li Q. Li H. Yao X. Wang C. Liu H. Xu D.. (2021). Stress response and hearing loss differentially contribute to dynamic alterations in hippocampal neurogenesis and microglial reactivity in mice exposed to acute noise exposure. Front. Neurosci. 15, 749925. 10.3389/fnins.2021.749925 34955715 PMC8692372

[B57] Liang Z. Li A. Xu Y. Qian X. Gao X. (2021). Hearing loss and dementia: a meta-analysis of prospective cohort studies. Front. Aging Neurosci. 13, 695117. 10.3389/fnagi.2021.695117 34305572 PMC8295986

[B58] Lin F. R. Metter E. J. O'Brien R. J. Resnick S. M. Zonderman A. B. Ferrucci L. (2011). Hearing loss and incident dementia. Arch. Neurol. 6, 214–220. 10.1001/archneurol.2010.362 21320988 PMC3277836

[B59] Liu W. Johansson Å. Rask-Andersen H. Rask-Andersen M. (2021). A combined genome-wide association and molecular study of age-related hearing loss in H. sapiens. BMC Med. 19, 302. 10.1186/s12916-021-02169-0 34847940 PMC8638543

[B60] Liu Y. Fang S. Liu L. Zhu Y. Li C. Chen K.. (2020). Hearing loss is an early biomarker in APP/PS1 Alzheimer's disease mice. Neurosci. Letters 717, 134705. 10.1016/j.neulet.2019.134705 31870800 PMC7004828

[B61] Livingston G. Huntley J. Sommerlad A. Ames D. Ballard C. Banerjee S.. (2020). Dementia prevention, intervention, and care: 2020 report of the lancet commission. Lancet 396, 413–446. 10.1016/S0140-6736(20)30367-6 32738937 PMC7392084

[B62] Loughrey D. G. Kelly M. E. Kelley G. A. Brennan S. Lawlor B. A. (2017). Association of age-related hearing loss with cognitive function, cognitive impairment, and dementia: a systematic review and meta-analysis. JAMA Otolaryngol. 144, 115–126. 10.1001/jamaoto.2017.2513 29222544 PMC5824986

[B63] Lyu A. Kim T. H. Park S. J. Shin S. Jeong S. Yu Y. N.. (2020). Mitochondrial damage and necroptosis in aging cochlea. Int. J. Mol. Sci. 21, 2505. 10.3390/ijms21072505 32260310 PMC7177801

[B64] Maharani A. Dawes P. Nazroo J. Tampubolon G. Pendleton N.; Sense-Cog WP1 group (2018a). Visual and hearing impairments are associated with cognitive decline in older people. Age Ageing 47, 575–581. 10.1093/ageing/afy061 29697748

[B65] Maharani A. Tampubolon G. Dawes P. Nazroo J. Pendleton N. (2018b). Hearing aid use and cognitive function in older adults. J. Am. Ger. Soc. 66, 1130–1136. 10.1111/jgs.15363 29637544

[B66] Mahmoudi E. Basu T. Langa K. McKee M. M. Zazove P. Alexander N. (2019). Can hearing aids delay time to diagnosis of dementia, depression, or falls in older adults? J. Am. Ger. Soc 67, 2362–2369. 10.1111/jgs.16109 31486068

[B67] Mamo S. K. Reed N. S. Price C. Occhipinti D. Pletnikova A. Lin F. R.. (2018). Hearing loss treatment in older adults with cognitive impairment: a systematic review. J Speech Lang Hear Res. 61, 2589–2603. 10.1044/2018_JSLHR-H-18-0077 30304320 PMC6428235

[B68] Marinelli J. P. Lohse C. M. Fussell W. L. Petersen R. C. Reed N. S. Machulda M. M.. (2022). Association between hearing loss and development of dementia using formal behavioural audiometric testing within the Mayo Clinic Study of Aging (MCSA): a prospective population-based study. Lancet 3, e817–e824. 10.1016/S2666-7568(22)00241-0 36410368 PMC9831680

[B69] McFadden S. L. Ding D. Burkard R. F. Jiang H. Reaume A. G. Flood D. G.. (1999b). Cu/Zn SOD deficiency potentiates hearing loss and cochlear pathology in aged 129, CD-1 mice. J. Comp. Neurol. 413, 101–112. 10464373

[B70] McFadden S. L. Ding D. Reaume A. G. Flood D. G. Salvi R. J. (1999a). Age-related cochlear hair cell loss is enhanced in mice lacking copper/zinc superoxide dismutase. Neurobiol. Aging. 20, 1–8. 10.1016/S0197-4580(99)00018-4 10466888

[B71] McFadden S. L. Ding D. Salvi R. (2001). Anatomical, metabolic and genetic aspects of age-related hearing loss in mice. Audiology 40, 313–321. 10.3109/00206090109073128 11781044

[B72] Mitchell B. L. Thorp J. G. Evans D. M. Nyholt D. R. Martin N. G. Lupton M. K. (2020). Exploring the genetic relationship between hearing impairment and Alzheimer's disease. Alzheimer's Dement. 12, e12108. 10.1002/dad2.12108 33005726 PMC7517507

[B73] Mohammed A. Gibbons L. E. Gates G. Anderson M.L. McCurry S. M. McCormick W.. (2022). Association of performance on dichotic auditory tests with risk for incident dementia and Alzheimer dementia. Arch. Otolaryngol Head Neck Surg. 148, 20–27. 10.1001/jamaoto.2021.2716 34647974 PMC8517881

[B74] Mosnier I. Vanier A. Bonnard D. Lina-Granade G. Truy E. Bordure P.. (2018). Long-term cognitive prognosis of profoundly deaf older adults after hearing rehabilitation using cochlear implants. J. Am. Ger. Soc. 66, 1553–1561. 10.1111/jgs.15445 30091185

[B75] Nagtegaal AP, Broer L, Zilhao NR, Jakobsdottir J, Bishop CE. (2019). Genome-wide association meta-analysis identifies five novel loci for age-related hearing impairment. Sci. Rep. 9, 15192. 10.1038/s41598-019-51630-x 31645637 PMC6811684

[B76] Naylor G. Dillard L. Orrell M. Stephan B. C. M. Zobay O. Saunders G. H. (2022). Dementia and hearing-aid use: a two-way street. Age Ageing 51, afac266. 10.1093/ageing/afac266 36571777 PMC9792081

[B77] Nguyen M. Bonnefoy M. Adrait A. Gueugnon M. Petitot C. Collet L.. (2017). Efficacy of hearing aids on the cognitive status of patients with Alzheimer's disease and hearing loss: a multicenter controlled randomized trial. J. Alz. Dis. 58, 123–137. 10.3233/JAD-160793 28387664

[B78] Nyberg S. Abbott N. J. Shi X. Steyger P. S. Dabdoub A. (2019). Delivery of therapeutics to the inner ear: The challenge of the blood-labyrinth barrier. Sci. Transl. Med. 11, eaao0935. 10.1126/scitranslmed.aao0935 30842313 PMC6488020

[B79] O'Leary T. P. Shin S. Fertan E. Dingle R. N. Almuklass A. Gunn R. K.. (2017). Reduced acoustic startle response and peripheral hearing loss in the 5xFAD mouse model of Alzheimer's disease. Genes Brain Behav. 16, 554–563. 10.1111/gbb.12370 28133939

[B80] Paciello F. Rinaudo M. Longo V. Cocco S. Conforto G. Pisani A.. (2021). Auditory sensory deprivation induced by noise exposure exacerbates cognitive decline in a mouse model of Alzheimer's disease. eLife 10, e70908. 10.7554/eLife.70908.sa2 34699347 PMC8547960

[B81] Park S. Y. Kim M. J. Kim H. L. Kim D. K. Yeo S. W. Park S. N. (2018). Cognitive decline and increased hippocampal p-tau expression in mice with hearing loss. Beh. Brain Res. 342, 19–26. 10.1016/j.bbr.2018.01.003 29317248

[B82] Patel S. V. DeCarlo C. M. Book S. A. Schormans A. L. Whitehead S. N. Allman B. L.. (2022). Noise exposure in early adulthood causes age-dependent and brain region-specific impairments in cognitive function. Front Neurosci. 16, 1001686. 10.3389/fnins.2022.1001686 36312027 PMC9606802

[B83] Quaranta N. Coppola F. Casulli M. Barulli O. Lanza F. Tortelli R.. (2015). The Prevalence of peripheral and central hearing impairment and its relation to cognition in older adults. Audiol. Neurotol. 19, 10–14. 10.1159/000371597 25733360

[B84] Ren W. J. Liu Y. Zhou L. J. Li W. Zhong Y. Pang R. P.. (2011). Peripheral nerve injury leads to working memory deficits and dysfunction of the hippocampus by upregulation of TNF-α in rodents. Neuropsychopharmacology 36, 979–992. 10.1038/npp.2010.236 21289602 PMC3077267

[B85] Rigters S. C. Bos D. Metselaar M. Roshchupkin G. V. Baatenburg de Jong R. J. Ikram M. A.. (2017). Hearing impairment is associated with smaller brain volume in aging. Front. Aging Neurosci. 9, 2. 10.3389/fnagi.2017.00002 28163683 PMC5247429

[B86] Saji N. (2021). Association between hearing impairment and cognitive decline: the protective role of hearing aids. Brain Nerve. 73, 907–912. 10.11477/mf.1416201860 34376597

[B87] Sarant J. Harris D. Busby P. Maruff P. Schembri A. Dowell R.. (2019). The effect of cochlear implants on cognitive function in older adults: initial baseline and 18-month follow up results for a prospective international longitudinal study. Front. Neurosci. 13, 789. 10.3389/fnins.2019.00789 31427915 PMC6687844

[B88] Sarant J. Harris D. Busby P. Maruff P. Schembri A. Lemke U.. (2020). The effect of hearing aid use on cognition in older adults: can we delay decline or even improve cognitive function? J. Clin. Med. 9, 254. 10.3390/jcm9010254 31963547 PMC7020090

[B89] Sarant J. Z. Harris D. C. Busby P. A. Fowler C. Fripp J. Masters C. L.. (2022). No influence of age-related hearing loss on brain Amyloid-β. J. Alz. Dis. 85, 359–367. 10.3233/JAD-215121 34806606 PMC8842788

[B90] Sardone R. Battista P. Donghia R. Lozupone M. Tortelli R. Guerra V.. (2020). Age-related central auditory processing disorder, MCI, and dementia in an older population of Southern Italy. Otolaryngol Head Neck. Surg. 163, 348–355. 10.1177/0194599820913635 32312167

[B91] Segaux L. Oubaya N. Broussier A. Baude M. Canouï-Poitrine F. Naga H.. (2019). Identification of five frailty profiles in community-dwelling individuals aged 50–75: a latent class analysis of the SUCCEED survey data. Maturitas 127, 1–11. 10.1016/j.maturitas.2019.05.007 31351514

[B92] Shen Y. Hu H. Fan C. Wang Q. Zou T. Ye B.. (2021). Sensorineural hearing loss may lead to dementia-related pathological changes in hippocampal neurons. Neurobiol. Dis 156, 105408. 10.1016/j.nbd.2021.105408 34082124

[B93] Sonnet M. Montaut-Verient B. Niemier J. Hoen M. Ribeyre L. Parietti-Winkler C. (2017). Cognitive abilities and quality of life after cochlear implantation in the elderly. Otol. Neurotol. 38, e296–e301. 10.1097/MAO.0000000000001503 28806342

[B94] Sorrentino T. Donati G. Nassif N. Pasini S. Redaelli de Zinis L.O. (2020). Cognitive function and quality of life in older adult patients with cochlear implants. Int. J. Audiol. 59, 316–322. 10.1080/14992027.2019.1696993 31793801

[B95] Spicer S. S. Schulte B. A. (2005). Pathologic changes of presbycusis begin in secondary processes and spread to primary processes of strial marginal cells. Hearing Res. 205, 225. 10.1016/j.heares.2005.03.022 15953531

[B96] Sugiura S. Nishita Y. Uchida Y. Shimono M. Suzuki H. Teranishi M.. (2021). Longitudinal associations between hearing aid usage and cognition in community-dwelling Japanese older adults with moderate hearing loss. PLoS ONE 16, e0258520. 10.1371/journal.pone.0258520 34644353 PMC8513843

[B97] Sugiura S. Uchida Y. Nishita Y. Teranishi M. Shimono M. Suzuki H.. (2022). Prevalence of usage of hearing aids and its association with cognitive impairment in Japanese community-dwelling elders with hearing loss. Auris Nasus Larynx. 49, 18–25. 10.1016/j.anl.2021.03.017 33865654

[B98] Tai S. Shen C. Wang L. Chien C. (2021). Association of sudden sensorineural hearing loss with dementia: a nationwide cohort study. BMC Neurol. 21, 88. 10.1186/s12883-021-02106-x 33627087 PMC7904508

[B99] Van Camp G. Smith R.J.H. (2023). Hereditary Hearing Loss Homepage. Available online at: https://hereditaryhearingloss.org (accessed 2023).

[B100] Vella Azzopardi R. Beyer I. De Raedemaeker K. Foulon I. Vermeiren S. Petrovic M.. (2023). Hearing aid use and gender differences in the auditory-cognitive cascade in the oldest old. Aging Ment. Health 27, 184–192. 10.1080/13607863.2021.2007355 34937465

[B101] Viana L. M. O'Malley J. T. Burgess B. J. Jones D. D. Oliveira C. A. C. P. Santos F.. (2015). Cochlear neuropathy in human presbycusis: Confocal analysis of hidden hearing loss in post-mortem tissue. Hear. Res. 327, 78–88. 10.1016/j.heares.2015.04.014 26002688 PMC4554812

[B102] Vlajkovic S. M. Thorne P. R. (2021). Molecular mechanisms of sensorineural hearing loss and development of inner ear therapeutics. Int. J. Mol. Sci. 22, 5647. 10.3390/ijms22115647 34073285 PMC8198622

[B103] Völter C. Götze L, Haubitz, I. Müther J. Dazert S. Thomas J.P. (2021). Impact of cochlear implantation on neurocognitive subdomains in adult cochlear implant recipients. Audiol. Neurotol. 26, 236–245. 10.1159/000510855 33440376

[B104] Völter C. Götze L. Bajewski M. Dazert S. Thomas J.P. (2022a). Cognition and cognitive reserve in cochlear implant recipients. Front Aging Neurosci. 14, 838214. 10.3389/fnagi.2022.838214 35391751 PMC8980358

[B105] Völter C. Götze L. Kamin S. T. Haubitz I. Dazert S. Thomas J.P. (2022b). Can cochlear implantation prevent cognitive decline in the long-term follow-up? Front. Neurol. 13, 1009087. 10.3389/fneur.2022.1009087 36341108 PMC9631779

[B106] Vona B. Rad A. Reisinger E. (2020). The many faces of DFNB9: relating OTOF variants to hearing impairment. Genes 11, 1411. 10.3390/genes11121411 33256196 PMC7768390

[B107] Wang S. Wu C. (2015). Physiological and histological evaluations of the Cochlea between 3xTg-AD mouse model of Alzheimer's diseases and R6/2 mouse model of Huntington's diseases. Chin. J.Physiol. 58, 359–366. 10.4077/CJP.2015.BAD334 26717914

[B108] Wang S. Wu C. (2021). Tau phosphorylation and cochlear apoptosis cause hearing loss in 3xTg-AD mouse model of Alzheimer's disease. Chin. J. Physiol. 64, 61. 10.4103/CJP.CJP_79_20 33938816

[B109] Weible A. P. Stebritz A. J. Wehr M. (2020). 5XFAD mice show early-onset gap encoding deficits in the auditory cortex. Neurobiol. Aging 94, 101–110. 10.1016/j.neurobiolaging.2020.05.013 32599514 PMC7483957

[B110] Wells H. R. R. Freidin M. B. Zainul Abidin F. N. Payton A. Dawes P. Munro K. J.. (2019). GWAS identifies 44 independent associated genomic loci for self-reported adult hearing difficulty in UK Biobank. Am. J. Hum. Genet. 105, 788–802. 10.1016/j.ajhg.2019.09.008 31564434 PMC6817556

[B111] Whiteus C. Freitas C. Grutzendler J. (2014). Perturbed neural activity disrupts cerebral angiogenesis during a postnatal critical period. Nature 505, 407–11. 10.1038/nature12821 24305053 PMC3947100

[B112] World Health Organisation (2021). Deafness and Hearing Loss. Available online at: https://www.who.int/news-room/fact-sheets/detail/deafness-and-hearing-loss (accessed 2023).

[B113] Wu P. Z. Liberman L. D. Bennett K. de Gruttola V. O'Malley J. T. Liberman M. C. (2019). Primary neural degeneration in the human cochlea: evidence for hidden hearing loss in the aging ear. Neuroscience 407, 8–20. 10.1016/j.neuroscience.2018.07.053 30099118 PMC6369025

[B114] Xu W. Zhang C. Li J. Tan C. Cao X. Tan L. (2019). Age-related hearing loss accelerates cerebrospinal fluid tau levels and brain atrophy: a longitudinal study. Aging 11, 3156. 10.18632/aging.101971 31118310 PMC6555452

[B115] Zhou Y. Song J. Wang Y. Zhang A. Tan C. Liu Y.. (2019). Age associated variation in the expression and function of TMEM16A calcium activated chloride channels in the cochlear stria vascularis of guinea pigs. Mol. Med. Rep. 20, 1593–1604. 10.3892/mmr.2019.10423 31257512 PMC6625423

[B116] Zhuang H. Yang J. Huang Z. Liu H. Li X. Zhang H.. (2020). Accelerated age-related decline in hippocampal neurogenesis in mice with noise-induced hearing loss is associated with hippocampal microglial degeneration. Aging 12, 19493–19519. 10.18632/aging.103898 33041264 PMC7732316

